# Everybody's Page

**Published:** 1890-08-16

**Authors:** 


### Everybody's Page.

The negroes of Louisiana use the juice of the pine-apple as a cure for diphtheria.

Smelling salts should not be used persistently when a person faints; they may injure the lining membrane of the nose.

The Corporation of Eastbourne have initiated a system of granting certificates to lodging-houses in a sanitary condition.

Visitors at holiday resorts who are doubtful about the quality of the water, should put some in a bottle and leave it corked for a time. Then shake it thoroughly and remove the cork and smell. If there is the least musty or unpleasant odour have the water analysed before any more of it is drunk.

In the "Annals of Ulster" occurs the following ancient reference to the influenza:—A.D. 1326: "Awful thunder and lightning this year, which destroyed the corn and produce of Erinn, so that it was blanched and waste. An epidemial disease common throughout all Erinn which was called 'Slaedan' (prostration, influenza), which affected during three or four days every person, so that it was second only to death."

That "mony a little makes a muckle" is proved by the following story, which ought to lead to the endowing of many cots in our hospital wards.—"Buying a trifle one day in a shop in which was a post-office, the proprietor asked me if I did not care to keep the farthing change he gave me, if I would put it into a collecting box, as he was canvassing all the farthings he gave in change in order to endow a cot for a child. A year after I called and asked if he had made up the required sum and found he had done so."

When St. Ferdinand took Seville from the Moors he founded a leper hospital there, which still remains devoted to its original object. In the fifteenth century, so rigorous was the decree ordering all who were attacked with this terrible disease to be removed to this institution that several noble lepers, including two bishops, died within its walls. Even as late as the last century it was the custom for four patients to visit Seville daily, begging on horseback. As lepers were not allowed to speak to the inhabitants, they attracted attention by means of painted boards. The ancient hospital of St. Lazaro has undergone in its day many vicissitudes: but, thanks to the benevolence of individuals, it is reported to be now in a flourishing condition, its patients, who number from thirty to thirty-six, being tended by the Sisters of St. Vincent de Paul.

Poisoning is a terribly frequent crime in India; an analyst of Bombay last year investigated 170 cases of doubtful death, and found poison in 66 cases. In 33 of these the poison used was arsenic, in 20 opium, in five pounded glass, in three *dhatura*, in three mercury, and in two *nux vomica*. In one instance sweetmeat balls containing arsenic were found in the possession of a woman who had given directions that they were to be sent to certain persons with whom she was at enmity; and in another nine persons ate bread containing a large quantity of arsenious oxide. In one case a man mixed poison with his food and accused another of trying to poison him. A woman confessed to having pounded her glass bangles and mixed the powder with her husband's bread. Glass seems to be used for this purpose only when poisons are not within reach, and the attempts usually fail on account of the grittiness of the glass exciting suspicion. Arsenic, being easily obtainable, is the chief resource of the Indian poisoner, and is usually administered in sweetmeats.

As a rule, a sore or inflamed eye should not be bandaged; a shade should be worn.

Dr. Giaxa, of Pisa, says that the cholera and typhoid microbes are killed by whitewash, but not so the microbes of tuberculosis, typhoid, carbuncle, and other diseases.

Milan was the first Italian city in which cremation obtained the sanction of the Government, and the first body cremated there was that of Signor Albert Keller on January 22nd, 1876.

Some medical man should really inquire into the following peculiar case: A "grateful patient" writes to the patentee of some complexion wash—"For years I was troubled with a rough red skin, but the use of your wash has quite removed it." This "grateful patient" might sit as a model for the saint who was flayed alive.

Amongst the many classes liable to cancer according to theoretical physicians are meat eaters, vegetarians, spirit or beer drinkers, dwellers by water-side, and in other topographical conditions. No theory fits every case, but each case can fit a theory. In the meanwhile surgeons are becoming more skilful as cancer becomes more common. It is a race for life; may the surgeons win!

"One man's meat another man's poison," is a proverb we appreciate after studying books of travel. David Livingstone tells us that the aboriginal Australians and the Hottentots prefer the intestines of animals, and he adds that "it is curious that this is the part which wild animals always begin with, and that it is the first choice of our men." Humboldt saw children drag enormous centipedes from their holes and crunch them between their teeth; but insects and their larvæ are favourite foods in many parts of the world. In the West Indies a large caterpillar, found on the palm tree, is reckoned a great delicacy. Stanley has strange tales to tell of the food appreciated by the inhabitants of Central Africa, but this subject will be more scientifically dealt with in Surgeon Parke's forthcoming book.

Why is it a man cannot be ill gracefully and agreeably? It is not such a very hard fate to rest quietly in bed and be waited on hand and foot by one's family, and be fed on exquisite delicacies. Women take only too kindly to the rôle of an invalid; the sofa, the fleecy white shawl; the little cups of beef-tea, or plates of oysters. Once let a woman taste the dreamy pleasures of this sort of existence, and unless some shock or sense of duty arouses her, she will calmly continue for the rest of her days in the pleasant path before her. She smiles sweetly at the little attentions offered her; she dresses in the daintiest of semi-toilettes, and she looks so pretty and gentle and patient that it seldom dawns on her husband that the existence is an ignoble one. But let my lord fall ill, and oh dear! what a different tale to tell. The valet comes flying from the room followed by a boot; the cook gives notice because master called the beef-tea "beastly stuff;" the housemaid is in tears because she is not allowed to sweep or dust the sick-room. Man, noble man, is a pitiful object when he is sick. Get him thoroughly ill, and he is a better patient than a woman; all hospital nurses prefer the men's wards to the women's; but if he is merely laid up for a day or two with a cold or a bilious attack, he persistently kicks against the pricks, instead of wisely enjoying the rest which a beneficent nature has imposed on him. The pity is that men are not better, and women worse invalids; but perhaps this will be arrived at when the day dawns which shows the equality of the sexes.

